# Evaluation of pre-hospital use of a valsalva assist device in the emergency treatment of supraventricular tachycardia [EVADE]: a randomised controlled feasibility trial

**DOI:** 10.1186/s40814-020-00616-y

**Published:** 2020-05-25

**Authors:** Andrew Appelboam, Jonathan Green, Paul Ewings, Sarah Black, EVADE study SWASFT collaborators, Adam Bedson, Jonathan Brownett, Peter Caine, Alison Coppola, Victoria Davis, Alison Jones, Robin Jones, Nicola Greathurst, Tom Holgate, Natalie Kean, Andrew Phillips, Claire McKenning, Michael Rennoldson, Gemma Richards, Chloe Rowlinson, Mark Spurrell, Johnathon Storry, Gary Tennet, Paul Wade, Fiona Warren, Martin White, Craig Wilkins, Emma Wilkinson

**Affiliations:** 1 University of Exeter Medical School, St Luke’s, Exeter, Devon UK; 2 Academic Department of Emergency Medicine, Royal Devon and Exeter Hospital NHS Foundation Trust, Exeter, Devon UK; 3 South West Ambulance Service Foundation Trust, Abbey Court, Eagle Way, Exeter, Devon UK; 4 Faculty of Health, University of Plymouth, Devon, UK

**Keywords:** Supraventricular tachycardia, Valsalva manoeuvre, Emergency care, Valsalva assist device

## Abstract

**Background:**

The valsalva manoeuvre is an internationally recommended initial treatment for supraventricular tachycardia (SVT). The pre-hospital use of a valsalva assist device, to help deliver this manoeuvre, could improve cardioversion rates and reduce the need for patients to attend hospital.

**Methods:**

We conducted a randomised controlled feasibility trial comparing ambulance clinician use of a valsalva assist device versus standard care to treat adult patients presenting to an ambulance service in the south west of England. Eligible consenting participants were randomised 1:1 to device or standard care with trial procedures mirroring a proposed definitive trial.

Feasibility was assessed upon ambulance clinician and participant recruitment rates and feedback, data completeness and potential future primary outcome rates.

**Results:**

Over a 6 months period (1 July to 31 December 2018), 276 (23%) of 1183 eligible ambulance clinicians were trained and they recruited 34 participants; approximately 10% of patients presenting with suspected SVT during that time. Seventeen participants were randomised to each arm and all underwent their allocated valsalva strain method. All trial data and 63/68 (93%) of pre and post-valsalva ECGs were available. Seven (21%) participants had ineligible initial rhythms on retrospective expert ECG review. Valsalva assist device use was associated with cardioversion and non-conveyance in 4 (24%) and 2 (12%) participants respectively. No participants assigned to standard care were cardioverted and all were conveyed. Participant feedback highlighted the challenges of retaining trial information during an SVT attack.

**Conclusions:**

The trial achieved efficient clinician training, randomisation and data collection, and there was an encouraging effect signal associated with device use. However, trial design changes should be considered to address the relatively small proportion of eligible patients recruited and challenges identified with consent and confirmation of cardioversion as a primary outcome.

**Trial registration:**

The trial was registered with ClinicalTrials.gov (NCT03514628) on 2 May 2018.

## Background

Supraventricular tachycardia (SVT) is a heart rhythm disorder with an estimated incidence of 35 episodes per 100,000 persons per year [1]. Episodes of this tachyarrhythmia frequently affect otherwise healthy individuals, are unpleasant and disruptive to patients’ lives [2]. Patients frequently seek medical help during attacks of SVT with many being attended by ambulance services and subsequently conveyed to an emergency department (ED) [3] An Australian regional ambulance service saw over 800 episodes a year [4], and an (unpublished) service evaluation by the UK’s South Western Ambulance Service NHS Foundation Trust (SWASFT), suggests a similar number are seen by this service.

The valsalva manoeuvre (VM) is a safe, internationally recommended, first line physical treatment for SVT [5, 6]. It causes slowing of the heart mediated by the vagus nerve which can terminate attacks (cardioversion) [7]. If this can be achieved by patients themselves or by pre-hospital emergency clinicians, conveyance to hospital and unpleasant second line intravenous treatments (such as adenosine) might be avoided [4, 8, 9].

Although the VM has historically had a relatively low (5–27%) rate of cardioversion in clinical practice [10], recent studies have shown that higher cardioversion rates approaching 50% can be achieved using a postural modification (the modified VM) and a 40-mmHg, 15-s strain delivered with a modified sphygmomanometer [11, 12]. Such manometers are not routinely carried by ambulance services and they also could not realistically be given to patients for use out of hospital (attacks can recur and patients can perform their own VM).

Blowing into an empty syringe has been used as an alternative [13], but these are unreliable in providing correct and consistent pressures [14–16]. A valsalva assist device (VAD) [Meditech Systems Ltd] has recently been developed. It has been designed to provide the recommended resistance to exhalation at a pressure of 40 mmHg, is small and portable and can be conveniently packaged with clear instructions on how to correctly perform the modified VM [17]. If successful, this single patient use device could then be left with the patient, should an attack recur.

An evaluation of this device’s effect on cardioversion and conveyance rates and cost effectiveness in the pre-hospital setting compared to current techniques would require a large definitive trial [18]. To determine whether such a trial is feasible, we designed and conducted a randomised controlled feasibility study in a single UK ambulance trust. The specific objectives of the study were to assess the pre-hospital clinician training and enrolment rates, the randomisation and consent processes, participant recruitment rates, data collection, and the acceptability of device use by pre-hospital clinicians. The study was designed to mirror a definitive study and to capture key feasibility data.

## Methods

### Trial design

We conducted a pragmatic, randomised controlled feasibility trial, with an evaluation of trial processes, comparing VAD versus standard practice delivered VMs in patients with SVT presenting to an ambulance service in the south west of England (SWASFT). Recruitment of participants and trial interventions were carried out by trial-trained pre-hospital clinicians of participating ambulance stations. The trial was registered (NCT03514628), supported through NIHR research capability funding and sponsored by SWASFT. Ethical approval for the study was received from Oxford C REC (18/SC/0111)

### Selection of sites and clinician training

Expressions of interest were invited from the larger ambulance stations in the SWASFT region. Twenty-seven stations volunteered and were selected on the basis of their interest, size and/or proximity to a larger ‘hub’ station in case of vehicle redistribution between neighbouring stations. Station champions were selected to support training uptake and trial publicity. All eligible ‘lead’ ambulance clinicians (paramedics and ambulance technicians/equivalent) in participating stations were sent invitations to take part, together with an e-training package which included an overview of SVT management and the modified VM, the use of the VAD, proportionate Good Clinical Practice training and a summary video of trial procedures.

Previous paramedic studies in this region suggested approximately 8 clinicians per week might be recruited. A 10-week recruitment period would therefore enable an estimated 80 study-clinicians to be enrolled.

### Participant recruitment

Patients with suspected SVT presenting to trial-trained SWASFT clinicians were screened according to the following eligibility criteria over a 6-month period:

### Inclusion criteria

- Age 18 years and over
- Patient identified by the ambulance clinician as having SVT* eligible for treatment with the valsalva manoeuvre

* Regular, narrow complex tachyarrythmia with a QRS duration < 0.12 ms on ECG

### Exclusion criteria

- Unable or unwilling to give informed verbal consent
- Unstable condition (systolic blood pressure (BP) < 90 mmHg)
- Atrial fibrillation (AF) or atrial flutter on ECG
- Severe hypertension (systolic BP > 220 mmHg or diastolic BP > 120 mmHg)
- Contraindication or inability to perform a modified valsalva manoeuvre (any clinical reason in why patients could not perform a valsalva strain and or be laid flat and have legs lifted to 45°. For example, hypotension, haemodynamic instability, recent eye surgery, or hip problems preventing hip flexion).
- Third trimester pregnancy
- Prisoners
- Previous inclusion in the study

Eligible participants were given an initial verbal explanation of the study using a standard script as a guide and asked for their verbal consent to take part. Given the nature of the condition, the challenging setting for the research and the fact that the proposed intervention differed from standard practice only in the method used to generate the valsalva strain, we proposed that witnessed verbal consent was appropriate and proportionate [19]. The consent process used was carefully discussed with patient advocates and approved by an ethics committee experienced in emergency research. However, feedback on the feasibility and appropriateness of the consent process used was also an important aspect of this study.

### Randomisation and masking

Participants were randomised 1:1, with variable-sized blocks, to receive a standard care VM, as determined by SWASFT guidelines and at the treating clinician’s discretion (control) or VAD delivered VM (intervention) as their first treatment. The randomization schedule was prepared in advance by a statistician. Boxes, containing a VAD or ‘standard care’ notice, were prepared according to this schedule by independent volunteers.

Boxes were identical, opaque, masked with internal packaging to ensure they had the same weight and feel and closed with a tamper-evident seal. They were packaged with a screening aid memoir and a simple recruitment checklist/data collection form. Boxes were placed in all ambulance vehicles of participating stations, with the ECG leads carried on vehicles, for ease of access and only opened by a participating trial-trained clinician after an eligible participant had given their consent.

Ambulance stations were given a sufficient stock of sequentially numbered boxes to supply all front-line vehicles, with spares to support recruitment. Once a pack was used, ambulance staff would restock the vehicle on return to station with the lowest numbered box according to a strict log. Use of boxes was followed and audited by station leads and the research team. This process enabled effective randomisation and immediate availability of trial paperwork, without the need for telephone or computer use, and was specifically chosen to aid successful consecutive recruitment of this patient group in the pre-hospital setting.

Participants underwent a maximum of three valsalva manoeuvres at the discretion and direction of the treating crews according to trial training and SWASFT guidelines, using the VAD or usual strain method, e.g. 10 ml syringe according to trial allocation. Continuous ECG monitoring and recording of 12 lead ‘snapshot’ ECGs was available through standard SWASFT monitoring equipment (Ortivus/Physio Control). All other treatment and subsequent management was conducted at the discretion of the pre-hospital clinicians and according to national and local guidelines. Of note, SWASFT has a locally approved protocol for use of the modified valsalva and non-conveyance of well patients, if their SVT has cardioverted back to normal sinus rhythm.

After completion of paramedic treatment, participants were given further written information about the trial (including advice on what to do should their arrhythmia re-occur) and asked for confirmation that they were still willing to be included in the study. They were also offered the device used for their VM (e.g. syringe or VAD) to take away and asked if they would be willing to take part in a follow up phone call to seek their opinions about the research over the next 2 weeks. For consenting participants, a structured follow up call was conducted by a member of the research team.

### Sample size

A formal sample size calculation was not undertaken for this feasibility study in which recruitment rate was a primary outcome. However, based upon the projected paramedic training rate and SWASFT observational SVT incidence data, we estimated that we might expect to recruit and randomise approximately 20 patients with SVT to the study over a 6-month recruitment period. Both participant and paramedic recruitment were closely followed during the study to ensure recruitment and training strategies were effective.

Screening and notification of recruitment was taken from the pre-hospital clinician electronic patient care record (ePCR) which has a field to indicate involvement in a research program. Data collection, as far as possible, was designed to mirror normal procedures. Key source data were recorded on the randomisation box cover and its image captured in the ePCR (which has a camera facility) before being returned to the researchers in the internal SWASFT post.

Other source and outcome data were taken from the ePCR. Clinicians were asked to document return to sinus rhythm if noted on continuous ECG monitoring and to record pre- and post-VM ‘snapshot’ 12-lead ECGs on the ePCR. These were subsequently checked by an independent consultant emergency physician, blind to the crew’s trial allocation. All reported adverse events were followed up according to good clinical practice (GCP) principles [20].

### Statistical analysis

Analysis of results was conducted according to guidelines for feasibility studies, and the ConSORT framework extension for pilot and feasibility trials was used to report results [21]. The number of participating pre-hospital clinicians was reported as a number and percentage of those approached. Baseline data (e.g. demographics) were reported descriptively by group and outcome data as point estimates using suitable measures (e.g. number and %) in each group, with 95% confidence intervals. No formal comparison between groups was undertaken.

### Outcome measures

The primary outcome measure for this feasibility study was the rate of pre-hospital clinician training and participant recruitment. Other outcome measures included the views of participants about the consent process and other research procedures and the completeness of data. Potential primary outcomes in a definitive study would be return to sinus rhythm as determined by the ambulance post-VM 12 lead ECG or the rate of non-conveyance to hospital. The feasibility of collecting ECGs and data regarding conveyance and other emergency pre-hospital treatments was also assessed.

### Patient and public involvement

Patient and public testimonies about emergency treatment for SVT [22] have driven trials looking for better non-drug treatment of SVT. Patient involvement with our previous research and clinical experience has demonstrated that an effective valsalva manoeuvre, with avoidance of drugs such as adenosine, is associated with high patient satisfaction and convenience [10].

A local patient and public group (Exeter 10,000) have previously given an opinion on the concept and rational for the VAD and were supportive of future trials into its use [17]. Patients and patient advocates were further involved in the design and conduct of this project, particularly in respect of the consent process and in the trial management group.

Feedback from participants on their experience of the research elements in this pilot was also sought through an invitation to participate in a phone call follow-up (maximum of 3 attempts to contact). Participants consenting to this follow-up underwent a short series of structured questions about their experience, with further comments invited and an opportunity to attend a face-to-face focus group with researchers. Participant views are vital as to the acceptability of any proposed future definitive trial design.

## Results

### Pre-hospital clinician training and recruitment rates

The study was conducted between 1 July and 31 December 2018. Training was offered to 1183 eligible ambulance clinicians in the 27 participating stations. At this time, SWASFT employed around 2290 ‘lead’ ambulance clinicians based at 95 stations. As larger stations were chosen in preference, training was therefore offered to approximately 52% of all eligible SWASFT clinicians in this feasibility trial. A total of 276 ambulance clinicians took up training and were enrolled to recruit patients presenting with SVT to the trial. This represented approximately 23% of the eligible clinicians at the participating stations and 12% of all SWASFT ‘lead’ clinicians. Almost all of the enrolled clinicians were trained within the first 3 months of the trial.

### Participant recruitment and baseline characteristic

**Fig. 1:**
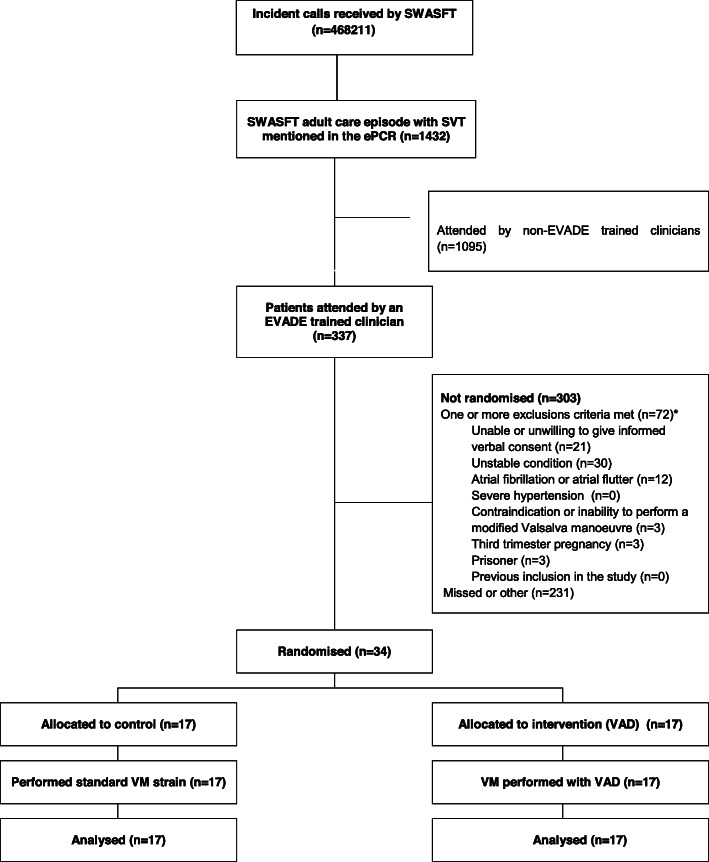
Consort diagram. SWASFT South Western Ambulance Service NHS Foundation Trust, ePCR Electronic patient record, SVT supraventricular tachycardia, VM valsalva manoeuvre, VAD valsalva assist device. *Proportion of reasons extrapolated from a detailed review of one month’s attendances

Over the 6 months duration of the study, 468211 incident calls were received by SWASFT. Of these, 1432 cases had ‘SVT’ or ‘supraventricular tachycardia’ mentioned in the electronic patient record (ePCR) and on review, suspected SVT was felt to be associated with the presenting complaint in approximately 330 of these cases. From these cases, 34 (10%) participants were attended and recruited by trial-trained ambulance clinicians. See Fig. 1.

Steady state recruitment, after the majority of staff were trained was approximately 10 participants recruited per month. Thirty clinicians recruited at least 1 participant, with two clinicians recruiting 2 participants and one recruiting three participants. Fourteen of the 27 stations recruited at least 1 participant (range 1–6). Given the small numbers involved, the groups were reasonably matched at baseline for age, sex and past history. See Table 1.

**Table 1:** Participant baseline characteristics

	Intervention	Control
Age (mean; median; range)	63; 68; 19–84	62; 62; 20–91
Sex (M:F)	10:7	7:10
Evidence concurrent illness	3	8
Previous symptoms of SVT but not documented or diagnosed	4	1
Previous documented SVT	6	5
Previous ablation therapy	1	0
Ischaemic heart disease	5	3
Diabetes	0	2
Hypertension	4	5
Valvular heart disease	0	1

*SVT* supraventricular tachycardia

### Randomisation and interventions

All 34 participants gave verbal consent and were randomised. Seventeen participants were randomised to intervention (VAD) and 17 to control. All participants underwent at least one VM attempt (range 1–3). Of the participants randomised to VAD, all received a VAD delivered VM. For the 17 participants randomised to control, a strain device in the form of a syringe was used for all VMs (5 ml size syringe for one participant; 10 ml for seven participants; 20 ml for eight participants; and 50ml for one participant). See Table 2 for details of interventions delivered.

**Table 2:** Trial Procedures and outcomes

	Intervention (*n* = 17)	Control (*n* = 17)
VAD delivered VM, *n*	17	0
Control delivered VM, *n*	0	17
Mean number of VM attempts, *n* (range)	2.3 (1–3)	2.7 (1–3)
Modified VM used, *n*	14	13
Legible pre-VM ECG available, *n* (%)	16 (94)	15 (88)
Legible post-VM ECG available, *n* (%)	16 (94)	16 (94)
Cardioversion, *n* (%, 95%CI)*	4 (24, 10 to 47)	0 (0, 0 to 18)
Not conveyed to hospital, *n* (%, 95%CI)	2 (12, 3 to 34)	0 (0, 0 to 18)
Total mean (median, range) ambulance episode duration (min)	62 (54, 27 to 117)	58 (61, 29 to 102)
Consent for phone follow-up, n	16	13
Phone follow up completed, *n*	12	9
Eligible presenting rhythm+, *n*	14	13
ECG Evidence of cardioversion on post-VM ECG in eligible participants+, *n* (%)	6 (43%)	3 (23%)

*VAD* valsalva assist device, *VM* valsalva manoeuvre, *ECG* electrocardiograph, *CI* confidence interval

*As recoded by the recruiting ambulance clinician

^+^As determined by independent retrospective review of ECGs by the emergency department consultant

### Cardioversion and non-conveyance rates

Of the 34 participants, all 17 control participants remained in SVT with a cardioversion rate of 0% (95% CI 0–18%). Use of the VAD in the 17 intervention group participants was associated with cardioversion in 4 participants, 24% (95% CI 10% to 47%). The use of a modified VM (mVM) was similar between the groups with 27 participants (14 intervention, 13 control) undergoing the mVM as part of their treatment.

Fifteen of the 17 intervention participants and all of the 17 control participants were conveyed to hospital. This represents a non-conveyance rate (95% CI) in intervention and control participants of 12% (3% to 34%) and 0% (0% to 18%) respectively.

### Data completeness

The method of return of data collection forms was by post only for five participants, by ePCR photo only for two participants and by both methods for 27 participants. Between the two methods of data collection, all data collection forms from the randomisation boxes were available for analysis. 63/68 (93%) of trial ECGs (31 pre-VM and 32 post-VM) were available and sufficiently legible (3 pre-VM and 1 post-VM ECGs had severe electrical interference and 1 post-VM ECG was not recorded) to allow independent analysis.

### Independent ECG review

On retrospective independent review of ECGs, 7 (21%) participants (4 control group, 3 intervention group) were judged to have an ineligible initial rhythm on the pre-VM ECG (2 AF, 1 broad complex tachycardia and 4 sinus tachycardia) and should have been excluded based upon the trial eligibility criteria. None of these participants achieved cardioversion and all were conveyed to hospital. Post-VM ECG evidence of cardioversion was independently confirmed in 3 of the 4 participants that were recorded by the ambulance clinician as having achieved VM-induced cardioversion (one participant’s SVT had recurred prior to the ECG). An additional 6 cases were judged to have sinus rhythm shown on the post-VM ECG after the ambulance clinician had recorded that SVT had continued after the VM (3 in each group). These cases are likely to represent later spontaneous cardioversion. Overall, the presence of sinus rhythm on a post-VM ECG in those judged to have had an eligible initial ECG rhythm on independent review was therefore 6/14 (43%) and 3/13 (23%) in intervention and control groups respectively.

### Adverse events

There were no serious adverse events reported. Dizziness was recorded in one intervention participant and a non-epileptic seizure occurred in one control participant (who had a known history of frequent non-epileptic seizures). Both fully resolved without sequelae.

### Participant and clinician feedback

Twenty-nine of the 34 participants consented to the follow-up telephone call and 21 (62%) participants (12 interventions, 9 controls) were contactable. The responses are summarised in Table 3. In the structured questions, participants felt that they had been given a clear verbal explanation for the trial, that they had an opportunity to ask questions and that they were generally satisfied with the consent process. Most participants reported being able to take in the trial information and that it was easy to decide to take part. However, fewer participants reported reading or recalling the take home information, and in the informal comments, over half those followed up, reported limited recall or ability to take in information at the time of their presentation. This finding was supported in the subsequent face-to-face focus group where participants highlighted the challenges around the transfer and retention of information during this acute illness. Despite this, all attendees felt that it had been appropriate to have been asked to take part and were pleased that they had had the opportunity to be involved in this research.

**Table 3:** Participant feedback phone follow-up

	Intervention	Control	Total
Trial verbal information was clear (agree/neither/disagree)	11/1/0	8/1/0	19/2/0
Opportunity to ask questions (agree/neither/disagree)	10/2/0	9/0/0	19/2/0
Consent process (satisfied/neither/dissatisfied)	11/1/0	9/0/0	20/1/0
Able to take in trial information (agree/neither/disagree)	9/2/1	8/1/0	17/3/1
Able to understand home information (agree/neither/disagree)	6/6/0	0/8/1	6/14/1
Deciding to take part (Easy/neither/difficult)	11/1/0	9/0/0	20/1/0

Thirty-seven (13%) clinicians involved in the study responded to an online survey sent to all those who had undertaken trial training. Most of this group felt the consent process used was appropriate or highly appropriate, that training was sufficient and all but one clinician (who was indifferent), were highly supportive of a definitive trial. Data collection and ECG capture were felt to be manageable in the context of recruiting to the trial during the routine care of their patients.

## Discussion

We have conducted a feasibility trial to assess clinician enrolment and participant recruitment rates to a study designed to determine the effects of a device on ambulance cardioversion and conveyance rates in patients with SVT.

Overall clinician and participant recruitment numbers exceeded our estimates which were based upon previous ambulance study training rates. Training and study procedures were designed to be straight forward. These were covered in an on-line training package and promoted by local ambulance station champions. We believe this contributed to the high uptake of clinician enrolment with nearly a quarter of eligible station clinicians taking part. For comparison, this is a similar clinician recruitment rate to that achieved during the AIRWAYS-2 pre-hospital trial, for which SWASFT was a trial site, but which required face-to-face training and took more than four times longer to achieve [23].

The rate of patient recruitment however was approximately 0.12 participants per trained clinician which was less than our original projections. If training of all eligible staff was replicated in the other stations across the SWASFT area with the same clinician and participant recruitment rate, we might expect approximately 126 patients to be recruited to a definitive trial, of the same design, per year. (0.12 × (0.23 × 2288) × 2). Using a ‘steady state’ recruitment rate of 10 participants per month seen in the last 2 months of the study across all stations gives an estimate of 220 participants. However, both estimates are considerably lower than our screening data and a previous SWASFT activity analysis suggested (approximately 600 patients a year presenting to ambulance clinicians with SVT). The lower rate of recruitment might be due to the perceived or actual barriers of recruiting to a randomised controlled trial in this setting or the availability of trial-trained clinicians.

Either way, it is likely that to achieve the level of recruitment required for a definitive randomised controlled trial of a similar design, the study would need to be conducted in a number of large ambulance trusts. Alternatively, a trial embedded into routine care, such as a step-wedge design [24], might be able to recruit at a rate nearer the numbers seen in previous service evaluation. This would also reduce the potential for clinician and station participation biases, inherent in this feasibility study design. For practical resource reasons, generally larger and geographically clustered stations were chosen to participate in this study. Although these stations had similar conveyance rates to non-participating stations and used the same trust clinical guidelines, stratified random selection of stations would be recommended in a definitive trial.

The rates of cardioversion and their associated confidence intervals observed in our study population were consistent with those reported in pre-hospital practice which vary widely (4.4% to 57.1%) as most studies are small [10, 25]. Although the trial was not designed to detect a difference in outcomes between the groups, there was a signal to suggest a higher rate of cardioversion and non-conveyance in the participants assigned to VAD delivered VMs. The rate of modified VM was similar between the groups; however, we did not collect details of exactly how the modification was carried out. One of the benefits of a bespoke device is the accompanying instructions to support delivery of an evidence based modification and it is possible that more clinicians using the VAD delivered the correct sequence of postural modifications.

Despite the relatively small number of participants recruited in this feasibility trial, the trial allocation process was efficient with equal numbers assigned to intervention and control and, despite the challenges associated with conducting a pre-hospital randomised controlled trial [26], worked well on a practical level: packs were available for all participating clinicians and recruited patients and the system for data collection was reliable with all data collection forms being returned by post or electronically. Most data were available through the usual electronic patient record (ePCR) and so routine use of an electronic ambulance patient record would be an important requirement for participating sites in a definitive trial.

Not all ECGs, however, were captured and overall a post-VM ECG was missing or illegible in 7% of participants. This is an important consideration in determining the primary outcome of a definitive trial. Cardioversion, as determined by the treating clinician at the time, could be used but confirmatory ECG evidence would be desirable. However, as shown by our independent ECG reviews, these can still be potentially misleading due to recurrence or spontaneous resolution of the arrhythmia. Strict timing of the post-VM ECGs, e.g.1 min post-VM, is unlikely to be practical in the pre-hospital setting.

Non-conveyance would be a reliable and easier outcome to confirm but non-conveyance after cardioversion remains relatively low and may not be universally advocated among ambulance trusts.

Over a fifth of the participants had an ineligible rhythm on retrospective expert review of their initial ECG. The exclusion of non re-entrant type SVT such as AF and other tachycardias that mimic SVT can be challenging even for experts. In a study conducted by emergency physicians in hospital, 8% of recruited participants were found to have an ineligible rhythm [6] and ECG interpretation by paramedics in the pre-hospital environment is likely to be more challenging. This study was pragmatic; a key inclusion criterion being detection of an SVT that the treating clinician identified as eligible for a valsalva manoeuvre. There is no evidence that vagal manoeuvres are harmful if used in an attempt to treat these excluded rhythms in the absence of contraindications. However, as it is unlikely to be successful, inclusion of such ‘ineligible’ participants would need to be taken into account to ensure adequate power to detect a difference in cardioversion or conveyance rates.

Available feedback from both participants and clinicians were broadly supportive of the trial design and a future definitive trial. However, challenges for participants and clinicians associated with delivering a randomised clinical trial in this setting, such as the recruitment of sufficient clinicians and patients are substantial and are very unlikely to capture anywhere near the numbers of SVT patients treated in routine practice. In addition, the limitations on taking in trial information highlighted by the participant feedback should further inform approaches to the consent process in the design of a definitive trial.

A definitive trial would also need a health economic analysis to take into account the cost of using a VAD, over current cheap alternatives, and any subsequent emergency care. This would add cost to such a trial and be challenging to accommodate within a limited trial budget.

Careful consideration should therefore be made for changes to the design of this study to mitigate against these recruitment, consent and trial cost challenges. Methodologies such as a stepped-wedge design, allowing the recruitment of participants during controlled changes to routine care, might be one option to overcome such challenges.

## Conclusions

We have completed a pre-hospital randomised controlled feasibility trial of the use a VAD to treat SVT, compared to standard care. This has shown recruiting sufficient numbers of clinicians and participants to a definitive trial of the same design would be challenging and likely to require participation of several large ambulance trusts. Although the treatment allocation and data collection processes used in this feasibility study design were efficient and could support a definitive trial, issues associated with case identification, consent processes and potential primary outcome selection remain a challenge. Design modification should be considered to help overcome these challenges.

## Data Availability

The datasets used and/or analysed during the current study are available from the corresponding author on reasonable request.

## Abbreviations

ECG: Electrocardiogram
ED: Emergency department
ePCR: Electronic Patient Care Record
GCP: Good Clinical Practice
SVT: Supraventricular tachycardia
SWASFT: South Western Ambulance Service NHS Foundation Trust
VAD: Valsalva assist device
